# New Methodology for Evaluating Surface Quality of Experimental Aerodynamic Models Manufactured by Polymer Jetting Additive Manufacturing

**DOI:** 10.3390/polym14030371

**Published:** 2022-01-18

**Authors:** Razvan Udroiu

**Affiliations:** Department of Manufacturing Engineering, Transilvania University of Brasov, 29 Eroilor Boulevard, 500036 Brasov, Romania; udroiu.r@unitbv.ro; Tel.: +40-268-421-318

**Keywords:** additive manufacturing, polymers, material jetting, 3D printing, airfoil, aerodynamic model, design of experiments, surface roughness

## Abstract

The additive manufacturing (AM) applications have attracted a great deal of interest with regard to experimental aerodynamic studies. There is a need for a universal roughness scale that characterizes different materials used in aerodynamic research. The main purpose of this paper is identification of the potential of a material jetting AM process to produce accurate aerodynamic surfaces. A new methodology to evaluate the roughness of aerodynamic profiles (airfoils) was proposed. A very short-span wing artifact for preliminary tests and a long-span wing model were proposed for design of experiments. Different artifacts orientations were analyzed, maintaining the same surface quality on the upper and lower surface of the wing. A translucent polymeric resin was used for samples manufacturing by polymer jetting (PolyJet) technology. The effects of main factors on the surface roughness of the wing were investigated using the statistical design of experiments. Three interest locations, meaning the leading-edge, central, and trailing-edge zones, on the upper and lower surfaces of the airfoil were considered. The best results were obtained for a sample oriented at XY on the build platform, in matte finish type, with a mean Ra roughness in the range of 2 to 3.5 μm. Microscopy studies were performed to analyze and characterize the surfaces of the wing samples on their different zones.

## 1. Introduction

Additive manufacturing (AM), known also as 3D printing, represents a key technology in the implementation of Industry 4.0 [1], based on its ability to fabricate highly complex and lightweight components directly from computer-aided design (CAD) files, saving time, cost, and effort. Additive manufacturing’s applications have attracted interest within many fields, such as the transportation industry, health sector, energy sector, and consumer production.

Seven categories of AM processes are defined by ISO/ASTM 52900-15 [2] standard based on the different joining techniques of materials to make parts from 3D model data, as follows: vat photo-polymerization (VP), binder jetting (BJ), material extrusion (ME), material jetting (MJ), sheet lamination (SL), powder bed fusion (PBF), and directed energy deposition (DED). One of the most accurate AM processes is MJ [3]. Material jetting processes, which include polymer jetting (PolyJet) and multi-jet printing (MJM) technologies, can be defined as a technique that selectively deposits droplets of material and cured them onto a build platform.

The main materials types used in the seven individual AM processes described by AM standards are polymers, ceramics, metals, and composite materials. Polymers became very popular in AM being used in the most of the AM processes and targeting a variety of applications [4].

The AM applications for experimental aerodynamic studies have attracted much interest within the aerospace, automotive, and wind energy sectors. Thus, the aerodynamic parts obtained by AM are used for testing in a wind tunnel or as final components for UAVs (unmanned aerial vehicle), drones, wind turbines, and small aircrafts. The main requirements of an aerodynamic part are the light weight, a smooth surface, and good mechanical characteristics.

Surface roughness is an important factor in aerodynamics that can significantly influence the fluid dynamics and the heat transfer [5]. The roughness of the wing skin increases the skin friction drag, which is one of the parasite drag components [6]. Three factors cause the parasite drag of an aerodynamic vehicle (e.g., an aircraft): the aircraft’s shape, construction type, and material. Parasite drag is split into three types: form drag, interference drag, and skin friction drag. The skin friction coefficients are sometimes based on experimental data for flat plates with various amounts of roughness. An inhomogeneous surface roughness distribution on an unmanned aircraft wing after many hours of flight was determined in [7]. It was mentioned that the initial roughness of the wing manufactured by a classical method not by additive manufacturing was 2 μm. The anisotropic influence of the winds during the flight, over the wing geometry, and the interferences between fuselage and wing were factors that increased the roughness.

Preliminary studies about additive manufacturing by the PolyJet process of airfoils for aerodynamic tests were performed in [8], but the quality of the airfoil surface was not investigated. The experimental coefficient of lift and drag of a NACA 2412 airfoil made by selective laser sintering (SLS) technology was studied in [9], but the surface roughness study was not carried out. Olasek et al. [10] evaluated a symmetrical NACA0018 airfoil model made by different materials and 3D-printing methods and concluded that surface roughness influences the aerodynamics characteristics of the airfoil. They also mentioned that the surface roughness is low for multi-jet modeling (MJM), moderate for SLS, and high for fused deposition modeling (FDM), but a range of roughness values were not mentioned. The rotor blades of a wind turbine rotor 3D printed by FDM technology were tested in wind tunnel by [11], but the surface roughness characterization was not performed. A UAV model was developed and manufactured using the binder jetting process by Junka et al. [12] for wind tunnel testing. This aerodynamic model was built by plastic powder and binder and then post-processed in order to obtain a good quality surface, but roughness investigation was not performed. These works demonstrated that 3D printing significantly changes the approach to experimental aerodynamics.

Three main tasks are significant to evaluate AM systems and processes for standardization and implementation in the industry: the performance characterization of the AM processes, AM part characterization, and AM system capability [3]. The main test methods for AM parts characterization are focused on mechanical properties [13,14,15], surface aspects [16,17], and dimensional geometry requirements [18]. Based on artifacts or customized models [19,20,21], the performance of the AM process can be investigated. The basic characterization of an AM system can be achieved via geometric accuracy [20], surface finish [21], and minimum feature sizes of the artifact [19]. The standards related to AM do not dictate a specific measurement method of artifacts features [22].

The surface quality of the AM parts was investigated in many studies mainly focused on the surface roughness determination [21,23,24,25]. The main factor that affects the surface roughness in different AM processes is the deposition layer thickness. It was reported based on experimental study that components generated through material jetting technology have superior surface quality than material extrusion components [26]. Additive manufacturing processes that use very thin layers deposition reduce the surface roughness, improving the surface quality. PolyJet technology, using deposition layers of 16 μm [27,28], significantly reduce the surface roughness of the parts. Part orientation on the built platform influences the surface roughness of the AM part [29]. Many studies have investigated it for different AM processes. An optimal part orientation achieves good results [30]. In addition, the surface roughness of the 3D-printed parts can be affected by external factors. Thus, a wearing analysis about PolyJet parts was performed in [31].

Some studies examined the material properties’ characterization, process parameters, dimensional, and geometrical characterization of material jetting, but a lack of knowledge around the aerodynamic parts such as airfoils was found. There is also a need for a universal roughness scale that can describe every type of roughness for different materials used in aerodynamic studies. Thus, printing quality (surface roughness) is an important aspect when the parts are meant for the aerodynamic tests. Based on the AM standards, there is no general “best practice” to perform the measurements in AM, especially for aerodynamic surfaces (e.g., airfoils and wings).

The main aims of this article are to define a methodology for evaluating the surface quality of aerodynamic surfaces and to identify the potential of the material jetting AM process to produce accurate aerodynamic surfaces (e.g., airfoils and wings). A case study regarding polymer jetting process and its materials validates the proposed methodology.

## 2. Materials and Methods

**Figure 1:**
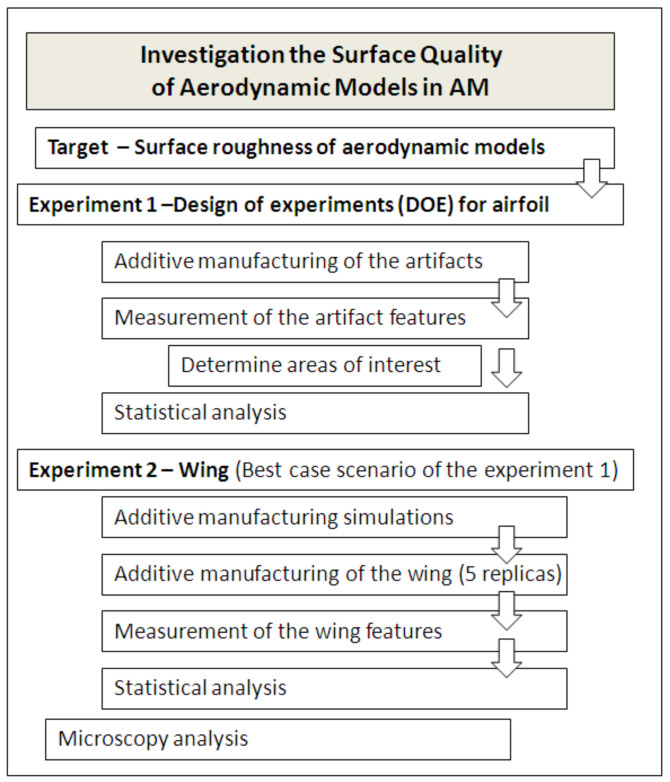
Flowchart of the proposed methodology of investigation of the surface quality of aerodynamic models produced by additive manufacturing.

The main objective of the proposed methodology is to evaluate the performance of an additive manufacturing process to produce aerodynamic artifacts. This methodology includes screening design of experiments (DOE) for a very short aerodynamic artifact and confirmatory experiments using a long aerodynamic artifact, which is followed by analysis and interpretation of the results, as is shown in Figure 1. The aerodynamic artifacts that were analyzed in this paper were airfoils and wings. Airfoil is a cross-sectional shape of an object whose motion through a fluid (e.g., air) is capable of generating significant lift force and a small drag force.

The target of the experiments is the surface roughness of the aerodynamic models obtained by an AM process. From aerodynamic considerations, the surface roughness of the upper and lower surface of the airfoil should have similar values. Therefore, the airfoil should be optimally positioned on the built platform to achieve it. In addition, the orientation of the part on the build platform influences the printing time and materials consumption. These parameters influence the total price of the 3D-printed part.

### 2.1. Aerodynamic Artifacts, Design of Experiments, and Simulations

**Figure 2:**
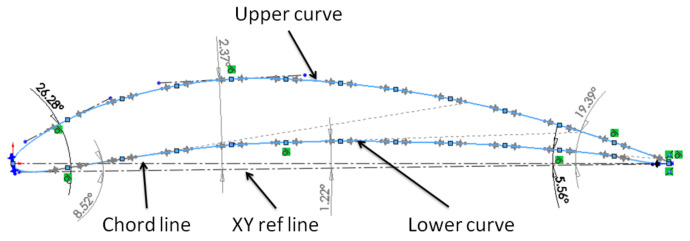
Terminology of asymmetrical airfoil curve—NACA 8410.

**Figure 3:**
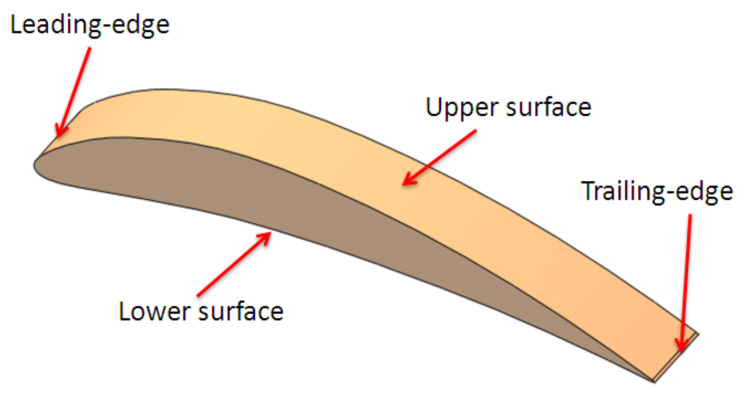
The very short-span wing model (airfoil).

**Figure 4:**
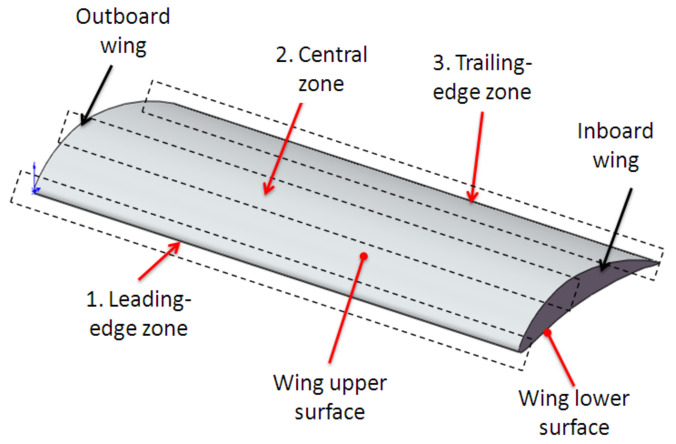
The long-span wing model.

Two types of aerodynamic artifacts were designed using the SolidWorks version 2016 software (Dassault Systèmes, Waltham, MA, USA), a very short-span wing (VS-SW) model denoted airfoil and a long-span wing (L-SW) model denoted wing. Both aerodynamic artifacts are designed using an asymmetrical airfoil such as NACA 8410 airfoil, with a chord length of 85 mm and taper ratio of 1 (Figure 2). Some basic terms related to airfoil are upper curve, lower curve, and chord line, as shown in Figure 2. The aerodynamic artifacts are designed with a span of 10 mm for the VS-SW model (Figure 3) and 200 mm for the L-SW model (Figure 4), respectively. The main characteristics that allow defining the locations on the wing are wing lower and upper surfaces, inboard and outboard of the wing, and three distinct zones: the leading edge, central, and trailing edge (Figure 4).

The VS-SW artifact is used for preliminary tests as screening design of experiments. Screening design of experiments is used to reduce a large set of factors, and usually, multiple replicates are not used. If a prediction model is searched for, using multiple replicates can increase the precision of the model. In this case, usually a minimum five replicas are used. In addition, the resources can dictate the number of replicates if the experiment is costly.

The preliminary experiment about the surface roughness investigation of the airfoil was designed [32] by choosing the control factors that affect the surface quality and their levels. There are many factors that affect the surface roughness in additive manufacturing [21]. The selection of the control factors depends on the particularities of the additive manufacturing process and the artifacts’ geometry. The following control factors that affect the surface quality were taken: airfoil orientation, surface finishing type, airfoil surface, and interest location. Details about the control factors and their levels are shown in Table 1.

**Figure 5:**
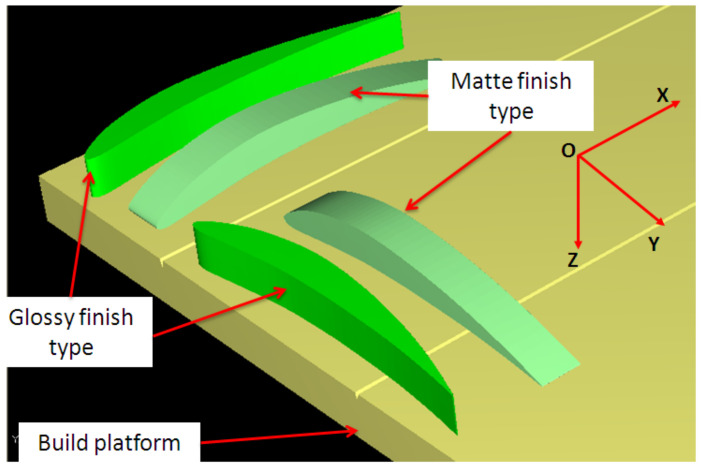
XY and YX orientations of the airfoil artifact. The first symbol indicates the longitudinal direction on the printing table and the second one indicates the transverse direction.

The airfoil orientation on the build platform is considered a two-level factor with basic orientations of parallel and perpendicular to the scanning direction (called XY and YX, respectively). The condition to keep the same surface quality on the upper and the lower surface of the airfoil was taken into consideration for airfoil orientations. The orientation of the AM build platform coordinate system was defined based on the ISO/ASTM 52921-13 standard [33]. The layout of artifact orientations on the build platform is shown in Figure 5.

Three interest locations for surface quality (roughness) investigations, meaning leading-edge, central, and trailing-edge zones, on the upper and lower surfaces of the airfoil were proposed based on aerodynamics considerations.

One distinctive factor of PolyJet technology is the surface finishing type. Matte and glossy finishing are the levels of finish type. A thin layer of support material is applied around the surface of the part in matte finish printing. It allows obtaining a uniform surface of the part. In the case of the parts printed in glossy finish type, the support material is deposited only on the bottom surfaces of the part, and the upper surfaces are glossy.

A general full factorial design with 24 factor combinations was performed to be able to investigate the influence of the control factors (Table 1) on the surface roughness of the airfoil. A statistical analysis of the data was performed for the airfoil, investigating and characterizing the effects of control factors and their interactions on the surface roughness of the airfoil. The analysis of variance (ANOVA) approach using a generalized linear model (GLM) was used. The statistical analysis was performed using the Minitab 17 software (Coventry, UK) [34]. The statistical indicators *p*-value and F-value determined in the ANOVA table indicate the significance of the results.

Based on the best-case scenario obtained from preliminary tests, a confirmatory experiment about roughness investigation was performed using the L-SW artifact. The span of this artifact is larger than the span of the VS-WS artifact. This larger wingspan is a factor that influences the manufacturing time of the wing in different orientation on the build platform.

Thus, some simulations of the AM process are required in order to minimize the manufacturing time and material consumption. The building time and the quantity of the model and support material were determined by simulation in Objet Studio software (Stratasys, Rehovot, Israel), as shown in Table 2. From the analysis of the simulation, the following conclusions can be drawn:

- The lowest building times were obtained in the case of XY matte and XY glossy orientation. The lowest time was obtained in the case of XY glossy, but different quality of the upper and lower surface of the airfoil was observed based on the support material influence on the lower surface.
- A medium time was obtained in the case of YX matte and YX glossy orientation.
- For the ZX and ZY orientation, a high building time was obtained, the highest being obtained for the ZY orientation.

**Figure 6:**
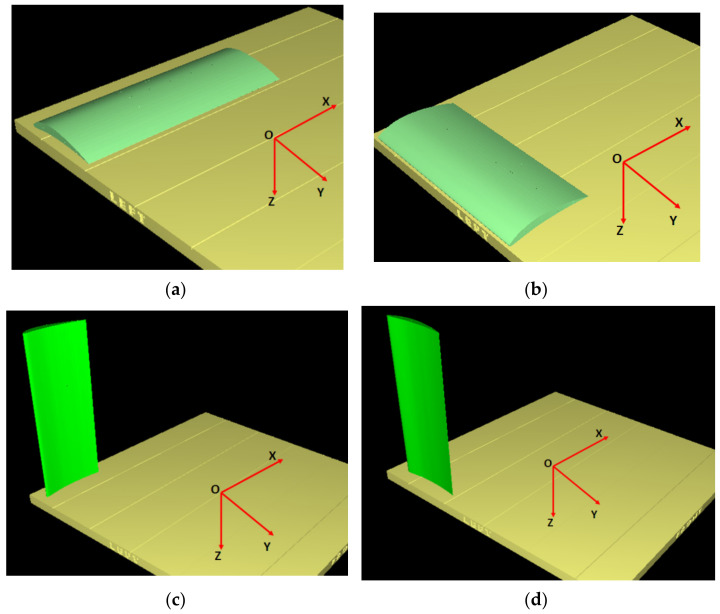
Simulations of the L-SW model in different orientation: (**a**) XY glossy; (**b**) YX matte; (**c**) ZX matte; (**d**) ZY matte.

Four orientations of the wing on the build platform (Figure 6) resulted to be candidates that can be taken into consideration, keeping the same quality of the upper and the lower surface of the wing.

Based on the simulations and the results obtained from the preliminary experiment (screening DOE), an optimal 3D-printing configuration for airfoils manufactured by PolyJet technology was determined to be XY matte. Five samples of wing in this optimal 3D-printing configuration were manufactured. The experimental surface roughness distribution on the long-span wing was analyzed.

### 2.2. Process Specification

All the samples (VS-SW and L-SW) were converted into standard triangle language (STL) files, imported into Objet Studio version 8.0.1.3 software (Stratasys, Rehovot, Israel), and manufactured using the Objet EDEN 350 PolyJet machine (Stratasys, Rehovot, Israel) [35]. The STL file conversion tolerances were set to a deviation of 0.01 mm and an angular tolerance of 4 degrees.

The materials known as FullCure 720 as model material and FullCure 705 as support material, supplied by Stratasys, were used to fabricate all samples. The composition of the Objet Fullcure 720 resin consists of acrylic monomer, urethane acrylate oligomer, epoxy acrylate, and photoinitiator. The main properties of Objet Fullcure 720 resin, also known as RGD720, are shown in Table 3 [36]. The support material, FullCure 705 resin, consists of acrylic monomer, polyethylene glycol 400, propane-1, 2-diol, glycerol, and photoinitiator. Diphenyl (2,4,6-trimethylbenzoyl) phosphine oxide is the photoinitiator used in UV treatment, as mentioned in [37]. The water contact angle of FullCure 720 material was investigated in [38], and it concluded that the Fullcure 720 is hydrophilic, with average contact angles of 81.0°. A Fourier transform infrared spectroscopy (FTIR) analysis of a material from the same acrylic family as FullCure720 was performed in [39]. They concluded that the spectrum shows that the material is acrylic based, (C=O) at 1721 cm^−1^.The chemical and physical characterization of polymers used in the PolyJet process will be investigated in a future work. The polymers characterization should follow a route as mentioned in [40].

The Objet EDEN 350 PolyJet machine works on polymer jetting technology that is derived from drop-on-demand (DOD) inkjet technology [41]. Basically, the process consists of depositing layers of resins that are 0.016 mm thick, which are leveled by a roller and hardened by ultraviolet (UV) light. The main PolyJet process parameters were temperature of around 72° Celsius of the print heads and the photopolymer resins and a vacuum of 6.2 atm applied in the print heads. The experiments were performed under a controlled laboratory temperature of 20° Celsius and relative humidity of 30%.

**Figure 7:**
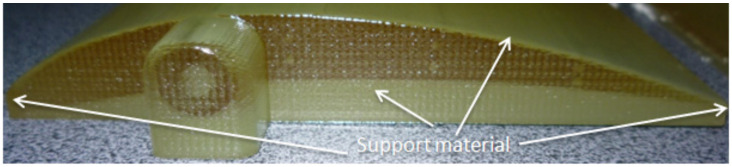
Specimen printed in matte finishing surrounded of support material.

Only the specimens printed in matte finishing (Figure 7) were post-processed by pressure water jet to remove the support material that surrounded the parts.

### 2.3. New Measurement Strategy for Surface Roughness of Airfoils

The roughness measurement strategy for an airfoil includes two tasks: establish the measurement areas of interest and apply filters (i.e., the cut-off length). The filters were chosen based on DIN EN ISO 4288 standard [42]. The Gauss-filtered measurements were set up as follows: an evaluation length of 12.5 mm and a cut-off value of 2.5 mm.

**Figure 8:**
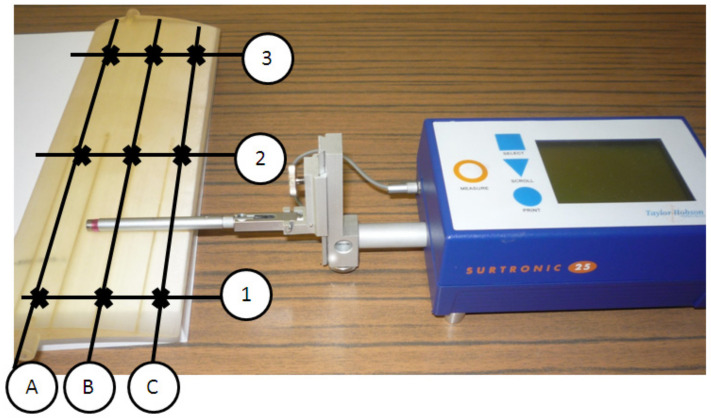
Measurement scheme of the airfoil roughness. Surface roughness measurement of the upper surfaces.

A new roughness measurement strategy of the wing is proposed, which consists of evaluating the surface roughness in three interest locations meaning leading-edge, central, and trailing-edge zone, on upper and lower surfaces of the airfoil (Figure 8). The interest locations were denoted “A” for the leading-edge zone, “B” for the central zone, and “C” for the trailing-edge zone, as is shown in Figure 8. In the case of the long-span wing, three sections denoted 1, 2, and 3 placed along the span wing were taken into consideration for the roughness measurements. One measurement section was considered for the VS-SW artifacts.

A Surtronic 25 contact surface roughness tester (Hoofddorp, The Netherlands) from Taylor Hobson was used to perform the measurements. The contact surface roughness tester was calibrated before performing the measurements. Profile measurements were repeated five times on each location of the airfoil surface, and the mean value was taken. The surface roughness Ra (the arithmetic mean deviation) was evaluated. The variability caused by the roughness measurement device was investigated, and data were processed within Minitab 17 software (Coventry, UK) using Gage R&R study [43].

A quality inspection through a microscopy study of the airfoils was performed using a Mitutoyo TM-1005 B optical-digital microscope (Mitutoyo, Kawasaki, Japan) with a digital micrometer head.

## 3. Results

The results of the performance of PolyJet process on the Objet EDEN 350 PolyJet machine to produce aerodynamic artifacts were analyzed taking into account the following considerations:

- The experimental surface roughness distribution on the upper and lower surface of the airfoil printed in two different quality modes and different orientations on the build platform;
- Surface quality issues of airfoil samples;
- The experimental analysis by microscopy of airfoils printed in different orientation;
- Results of statistical analysis.

All artifacts of the very short-span wing were manufactured in 2 h and 14 min, using 39 g of model material and 27 g of support material. The processing time for each long-span wing printed in XY orientation (best-case scenario) was 1 h and 38 min, and the consumption was 172 g of model material and 97 g of support material.

### 3.1. The Experimental Surface Roughness Analysis on Airfoils

**Figure 9:**
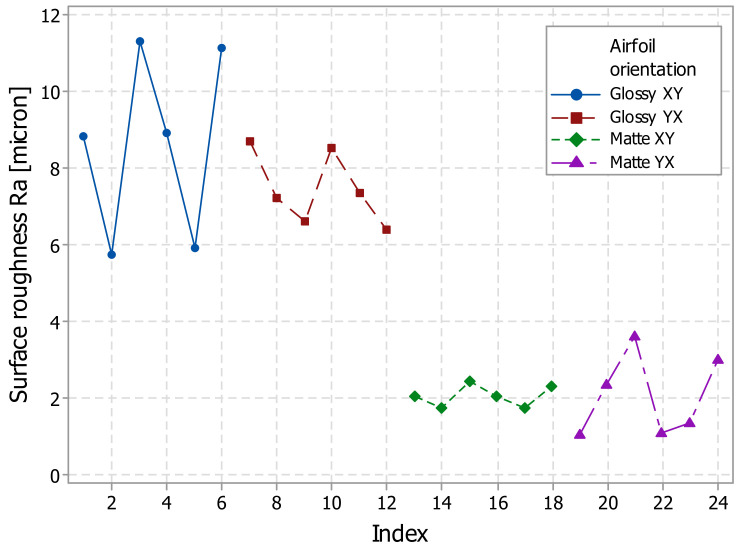
Surface roughness (Ra) distribution of the airfoils: glossy XY, glossy YX, matte XY, and matte YX.

Surface roughness distribution along the very short-span wing (airfoil) determined from experiments is shown in Figure 9.

The experimental roughness (Ra) values of the airfoil printed in matte finish were found in the range of 1.06 to 3.62 microns for the YX orientation and 1.74 to 2.46 microns for the XY orientation, as shown in Figure 9. The roughness of the artifacts printed in glossy finish presented higher values than the matte finish artifact, in the range of 5.72 to 11.3 microns for the XY orientation, and 6.4 to 8.68 microns for the YX orientations (Figure 9). Similar quality of the upper and lower surface of the airfoil was found in the mentioned range both for matte and glossy printed samples.

The lowest and relatively uniform roughness (Ra) was obtained for the airfoils printed in matte mode in the XY orientation. This is a reason why the L-SW experimental wings are printed in matte finishing mode.

### 3.2. Results of Statistical Analysis

The roughness tester variation based on the Gage R&R [43] study was much smaller than the variation of the surface roughness of the 3D-printed parts, proving the repeatability of the measurement system, as is mentioned also in [21].

From the ANOVA table (Table 4), the surface finishing type and the interest location are the significant factors that have a higher influence on the Ra roughness of the airfoils, taking into account their *p*-values. In addition, only the factor surface finishing type showed F_exp_ values greater than the critical F-value of 0.1% at α = 0.001. Thus, the results were significant at the 0.1% significance level. The percentage contributions ratios for all the factors are presented in Table 4. The most significant factor on the roughness parameter (Ra) was the surface finishing type, which explained 82.86% of the total variation. The next contribution on Ra came from the interest location, with a contribution of 4.46%. The airfoil orientation and airfoil surface factors have no important effect on roughness parameter (Ra), which is based on a higher *p*-values and low percentage contribution (PC%).

The evaluation of the influence of the control factors on the surface roughness (Ra) was performed through graphical analysis. The following graphs were obtained based on the statistical results, the main effects plot, interaction effects plot, and interval plot of Ra versus each factor.

**Figure 10:**
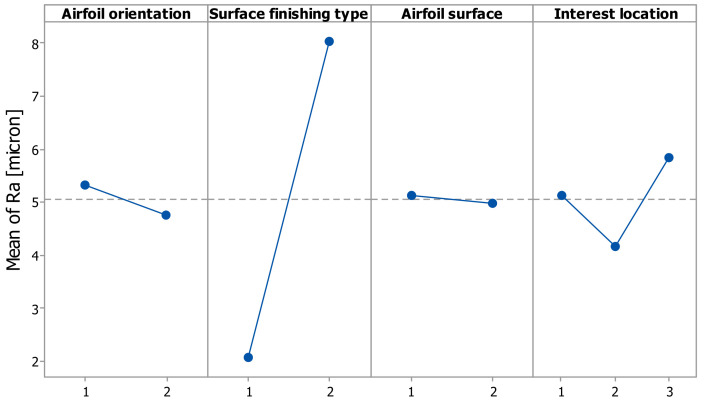
Main effects plot for surface roughness Ra.

**Figure 11:**
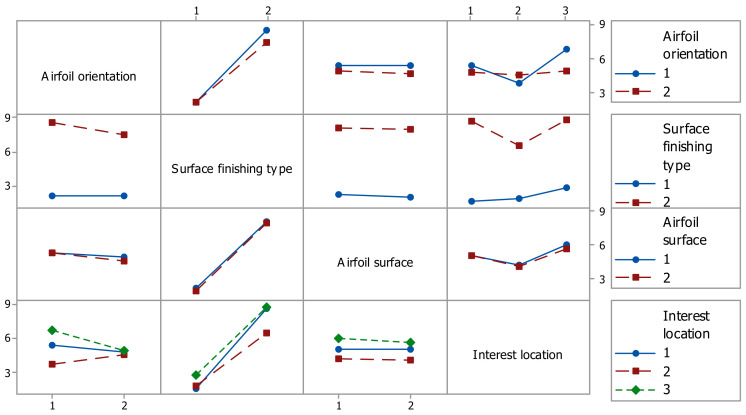
Interaction effects plot for surface roughness Ra.

The main effects for surface roughness were the airfoil orientation at level 1 (XY), the finish type at level 2 (glossy), and the interest location at level 3 (trailing-edge zone), as is shown in Figure 10. It is obvious that the factors surface finishing type, interest location, and their interaction had a significant influence on the surface roughness, as shown in Figure 10 and Figure 11.

**Figure 12:**
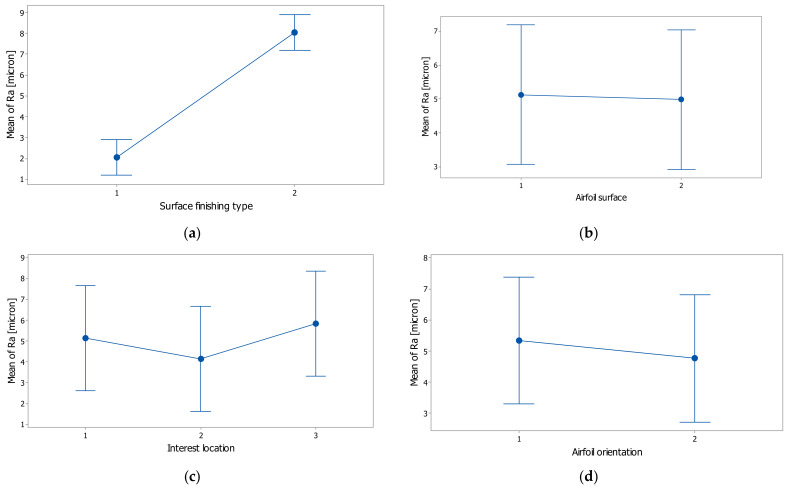
Individual standard deviations were used to calculate the intervals plot of surface roughness (Ra) versus (**a**) surface finishing type, (**b**) airfoil surface, (**c**) interest location, and (**d**) airfoil orientation; bars are standard errors of the mean.

The interval plots with standard error bars of each factor versus the roughness (Ra) are shown in the graphs from Figure 12. The difference between the means for Ra in the surface finishing type was significant because the interval bars did not overlap, as is shown in Figure 12a. While the means appear to be different, the differences for Ra in the airfoil orientation and airfoil surface were probably not significant because the interval bars easily overlapped (Figure 12b,d). The interest location (Figure 12c) had an influence on Ra, and it seems that at the central zone of the airfoil, the mean of Ra was lower, while for the leading-edge and trailing-edge zones, the mean was higher.

### 3.3. Tests Results about Long-Span Wing

The results of the statistical analysis of the L-SW data show that the coefficients of variation for all the wing regions are lower than 10%, which assures the data heterogeneity and expresses the repeatability of the experiments, as shown in Table 5. The coefficient of variation (CV) is a measure of spread that describes the variation in the data relative to the mean. The standard error of the mean estimates the variability between samples, whereas the standard deviation measures the variability within a single sample.

The highest values for the surface roughness of the wing were found on the leading edge of the airfoil. This can be explained taking into accord that the angle between the wing surface and the horizontal plane is around 25°, as confirmed by the reference [21]. The smallest Ra values are found on the central zone of the wing, which could be considered a near-horizontal surface.

**Figure 13:**
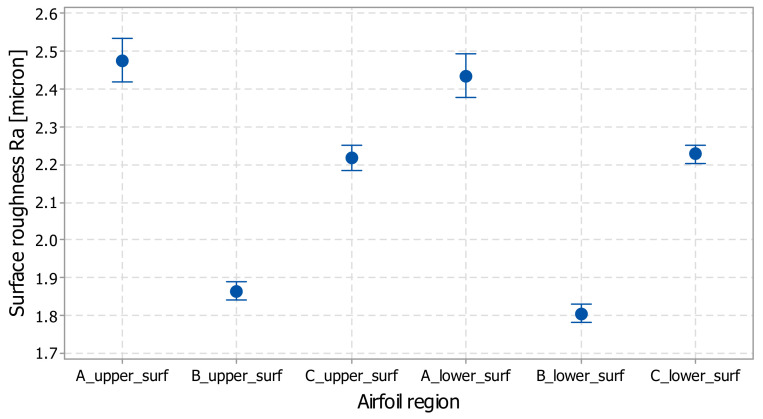
Interval plot of surface roughness for airfoil regions (matte XY orientation); bars are one standard error from the mean.

The interval plots of the surface roughness of the wing are in the range ±0.1 micron for all samples (Figure 13). Individual standard deviations were used to calculate the interval plot.

### 3.4. Quality Inspection of Airfoil Surface Based on Visual Inspection and Microscopy Analysis

Visual inspection and microscopy study were performed to analyze and characterize the surfaces of the samples in their different zones.

Surface errors on PolyJet-printed parts in glossy mode were determined in some studies [21,25]. Thus, rough surface areas were observed on the surface of the cylindrical parts printed on the Objet EDEN 350 perpendicular to the scanning direction [25]. In addition, horizontal steps marks [21] were visually observed on the flat faces oriented at 75° and 85° relative to the XOY plane. However, no visual flaws were detected on the vertical flat walls printed in the glossy style.

**Figure 14:**
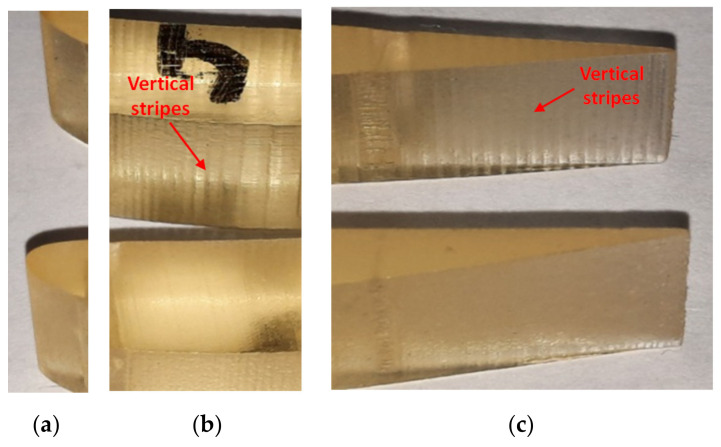
Comparative study of the airfoil surface in different interest locations for glossy XY (upper) and glossy YX (lower): (**a**) leading edge; (**b**) central zone; (**c**) trailing edge.

**Figure 15:**
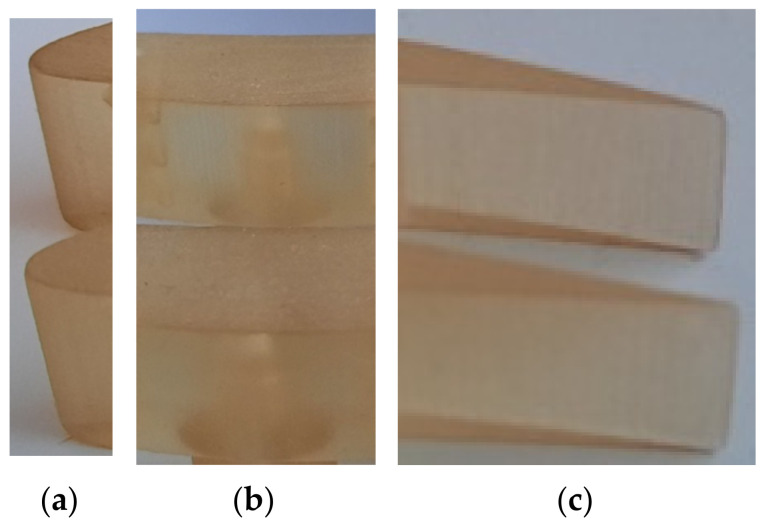
Comparative study of the airfoil surface in different interest locations for matte XY (upper) and matte YX (lower): (**a**) leading edge; (**b**) central zone; (**c**) trailing edge.

Based on visual inspection, two types of errors (rough surface areas) on the vertical walls of the airfoils (VS-SW) printed in glossy mode were determined (observed), as is shown in Figure 14. The first error consisting in vertical stripes on the airfoil surface was observed on the surface perpendicular on the scanning direction (*X*-axis direction) of the 3D printer. These were caused by the lower resolution of 0.042 mm in the X-direction and Y-direction compared to 0.016 mm in the Z-direction. The “vertical stripes” errors are predominant on the airfoil artifact printed in the XY orientation. The glossy sample printed in the YX direction presents a surface with a great density of points, which results in a homogeneous texture. There were no defects detected on the airfoils surface printed in matte mode, as is shown in Figure 15.

**Figure 16:**
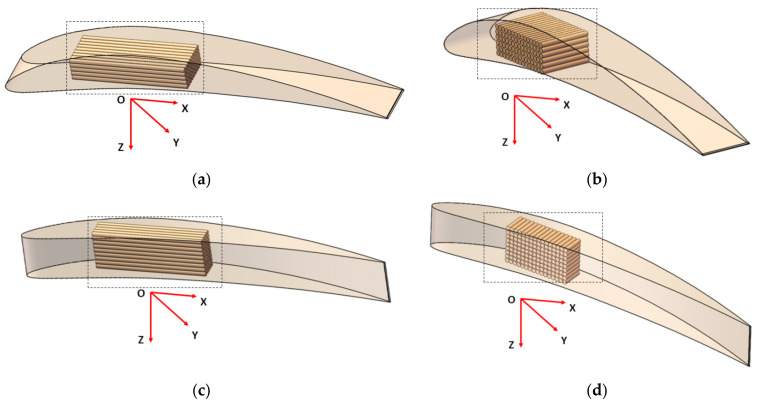
VS-SW specimens with partial views indicating the layers of cylinders for the different print orientations: (**a**) matte XY; (**b**) matte YX; (**c**) glossy XY; (**d**) glossy YX.

In the XY-direction orientation, fewer nozzles of the printer head are used compared to the YX orientation, as is shown in the partial views from Figure 16. Each nozzle deposits a train of droplets of resin grouped in a cylinder shape, and the level Z cylinders form a layer. Cylinders are similar to long fibers within composite materials. It can be seen that within the layers, the XY specimens have longer but fewer fibers than the YX specimens [14].

**Figure 17:**
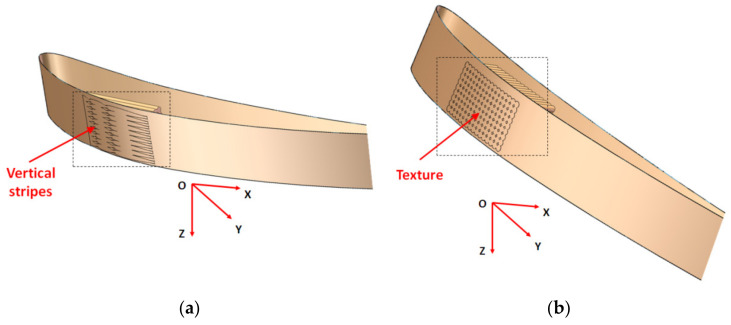
Theoretical texture of the airfoil surface: (**a**) glossy XY; (**b**) glossy YX.

There are more intersections between cylinders from the layers and airfoil surface in the YX orientation. In addition, each intersection could be approximated as a circular shape of very small size, which confers a homogeneous texture, confirming the experimental observations (Figure 17b). In the case of XY orientations, the intersections between simulated layers (e.g., the lateral surface of the cylinders) and airfoil surface leads to straight vertical stripes (Figure 17a). These vertical stripes are more pronounced in the glossy printed mode based on an increased UV exposure. In the matte finishing, a theoretical 3D model is difficult to be drawn because, an additional support material layer is deposited on the airfoil surface, which allows obtaining a uniform texture.

The microscopy analysis study was performed for quality inspection of airfoil surfaces. All the surfaces of the matte specimens are affected by the material support. Very small inclusions of the support material were detected on the surface of these specimens even after post-processing by cleaning with a pressure water jet.

**Figure 18:**
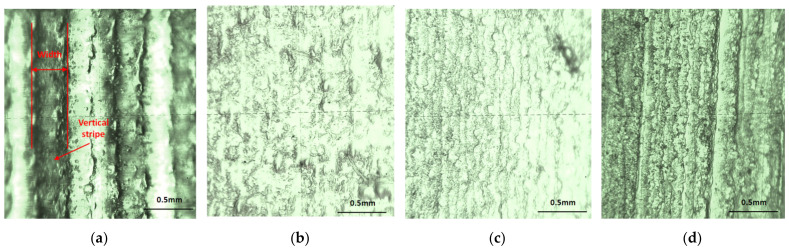
The texture of the airfoil surface: (**a**) glossy XY; (**b**) glossy YX; (**c**) matte XY; (**d**) matte YX.

The texture of matte specimens is shown in Figure 18c,d. Specimens printed in glossy mode present different kind of texture depending on their orientation. There can be seen a homogeneous airfoil surface for the glossy YX specimen (Figure 18). The width values of vertical stripes (Figure 18a) detected on the glossy XY specimen were in the range 0.213 to 0.386 mm, which were measured within the microscopy analysis.

**Figure 19:**
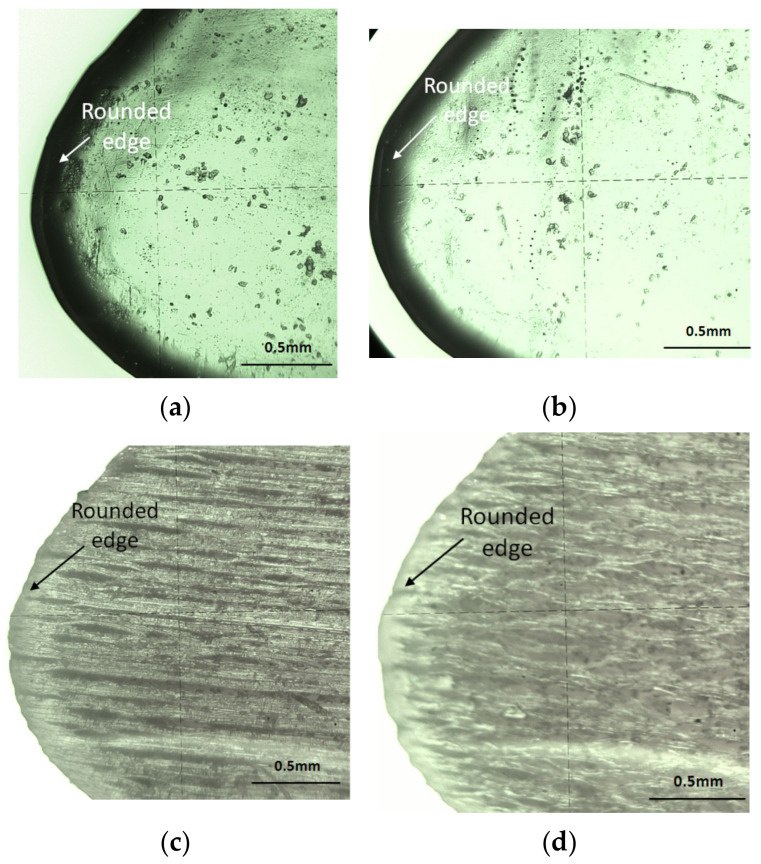
Lateral surface view of the airfoil on the leading-edge zone: (**a**) glossy XY; (**b**) glossy YX; (**c**) matte XY; (**d**) matte YX.

**Figure 20:**
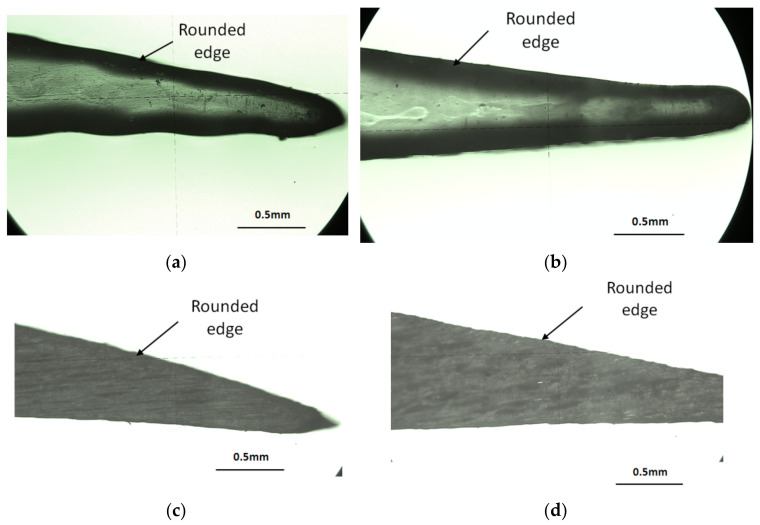
Lateral surface view of the airfoil on trailing edge zone: (**a**) glossy XY; (**b**) glossy YX; (**c**) matte XY; (**d**) matte YX.

Semi-transparent surfaces were detected for the specimens printed in glossy mode. The lateral shape of the airfoils was analyzed, especially on the leading edge and the trailing edge of the airfoil (Figure 19 and Figure 20). A rounded edge was detected around the airfoil curve. This is represented by a black border (Figure 19a,b and Figure 20a,b) in the case of glossy type. In addition, the edges of the airfoils printed in matte mode are rounded, as shown in Figure 19c,d and Figure 20c,d.

The smooth curve of the airfoil was detected for matte-printed specimens (Figure 19c,d and Figure 20c,d). The airfoil curve of glossy specimens presents some deviations from the theoretical profile in the lower part of the trailing edge, as is shown in Figure 20a.

## 4. Conclusions

There is a need for a universal roughness scale that can describe every type of roughness for different materials used in aerodynamic studies. This paper contributes to the characterization of the surface quality (roughness) of airfoils and wings made by the material jetting AM process. The new methodology based on aerodynamic interest locations and DOE can be used to characterize aerodynamic parts build by AM processes.

The following conclusions can be drawn:

- Material jetting is a quick and simple additive manufacturing process to produce aerodynamic models from polymers.
- The proposed methodology may assess the aerodynamic surface quality in a simple way based on a measurement scheme of roughness on airfoils.
- An inhomogeneous surface roughness distribution on an airfoil was obtained by PolyJet technology using an EDEN 350 system, which can be explained by different surface slopes on the airfoil zone such as the leading edge, central zone, and trailing edge.
- Based on preferential orientations on the build platform, a similar quality of the upper and lower surface of the airfoil was found, both for matte and glossy-printed samples. This could be a beneficial advantage for future aerodynamic studies.
- The experimental roughness (Ra) values of the airfoil printed in PolyJet matte finish were found in the range of 1.06 to 3.62 microns for the YX orientation and 1.74 to 2.46 microns for the XY orientation. The roughness of the airfoils printed in glossy finish presented higher values than matte finish airfoil, in the range of 5.72 to 11.3 microns for the XY orientation and 6.4 to 8.68 microns for the YX orientation.
- The disadvantage of the glossy finish includes some surface quality issues as rough surface areas on the airfoil surface, which were determined by visual inspection, microscopy, and theoretical studies.
- The most influential factor on airfoil surface roughness for the PolyJet process was surface finish type, which was determined from DOE investigation.
- Based on the simulations and the results obtained from the screening DOE, an optimal 3D-printing configuration for airfoils manufactured by PolyJet technology was determined to be XY matte. In addition, the microscopy studies showed that the airfoils printed in matte mode present a homogeneous surface.

Additional study could investigate the dimensional accuracy of the airfoils built by 3D printing, using quality control tools such as the 3D-scanning technique [44]. In addition, future research will investigate other AM processes and materials that can be candidates for airfoils and wings manufacturing based on the proposed methodology.

## Figures and Tables

**Table 1:** Control factors and their level.

Level	Target	Airfoil Orientation ^1^		Surface-Finishing Type		Airfoil Surface		Interest Location	
	Symbol	Symbol	Value	Symbol	Value	Symbol	Value	Symbol	Value
1	Ra	1	XY	1	Matte	1	upper	1	Leading-edge zone
2		2	YX	2	Glossy	2	lower	2	Central zone
3		-	-	-	-	-	-	3	Trailing-edge zone

^1^ Only the aerodynamic artifact orientations that allow obtaining a similar roughness distribution on the upper and lower surface of the airfoil were considered.

**Table 2:** Estimated 3D-printing parameters for different orientation on the build platform of the L-SW model.

Wing Orientation	Surface Finishing Type	Building Time (hour:min)	Model Consumption (g)	Support Consumption (g)
XY	matte	1:38	172	97
	glossy	1:34	170	81
YX	matte	3:16	177	102
	glossy	3:13	174	86
ZX	matte	10:59	187	99
	glossy	10:56	175	35
ZY	matte	22:46	215	127
	glossy	22:40	202	59

**Table 3:** Objet FullCure 720 properties [36].

Property	ASTM	Metric
Tensile Strength	D-638-03	50–60 MPa
Elongation at Break	D-638-05	15–25%
Flexural Strength	D-790-03	60–70 MPa
Rockwell Hardness	Scale M	73–76 Scale M
Water Absorption	D-570-98 24 h	1.5–2.2%
Polymerized Density	ASTM D792	1.18–1.19 g/cm^3^

**Table 4:** The percentage contribution ratio based on generalized linear model (GLM).

Source	DF	Seq SS	Seq MS	F_exp_	F_0.1%_	p	PC (%)
Airfoil orientation	1	1.955	1.955	1.15	15.37	0.299	0.07%
Surface finishing type	1	214.503	214.503	125.66	15.37	<0.001	82.86%
Airfoil surface	1	0.137	0.137	0.08	15.37	0.781	0.005%
Interest location	2	11.548	5.774	3.38	10.38	0.057	4.46%
Error	18	30.726	1.707				11.86%
Total	23	258.869					100%

**Table 5:** Statistics of wing surface roughness Ra for five samples.

Airfoil Region	Mean Roughness Ra [micron]	Standard Deviation [micron]	Coefficient of Variation [%]
A_upper_surf	2.47	0.225	9.08
B_upper_surf	1.86	0.089	4.82
C_upper_surf	2.22	0.131	5.9
A_lower_surf	2.43	0.221	9.09
B_lower_surf	1.80	0.090	5.02
C_lower_surf	2.22	0.094	4.25

## Data Availability

Not applicable.
